# Supplementary material for: Amino acid and viral binding by the high-affinity Cationic Amino acid Transporter 1 (CAT1) from Mus musculus
Source: Nat Commun. 2026 Feb 16;17:2829. doi: 10.1038/s41467-026-69421-0 (PMC13022406; doi:10.1038/s41467-026-69421-0)
Supplement: Supplementary file 4 — Source Data [file 41467_2026_69421_MOESM4_ESM.zip › Source Data/Saur_source data_gels.pdf]

a

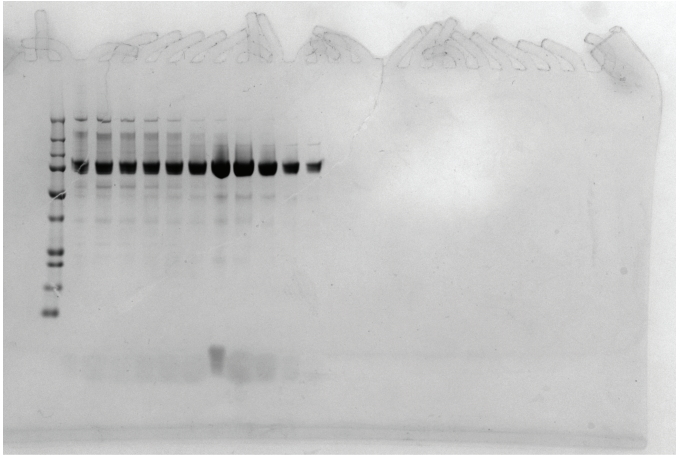

b

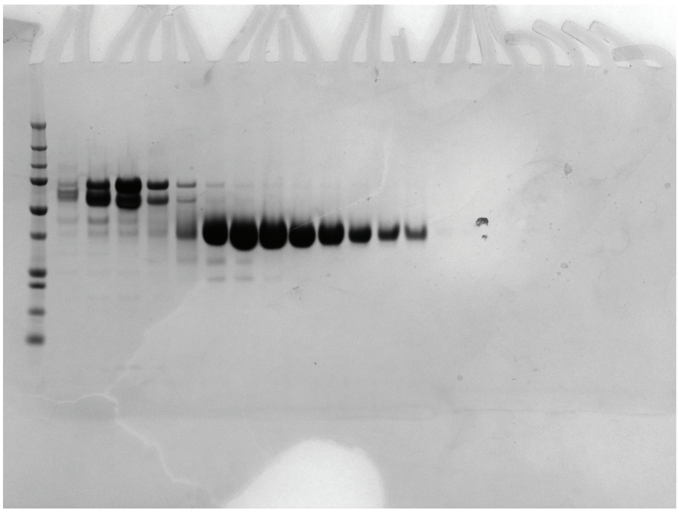

c

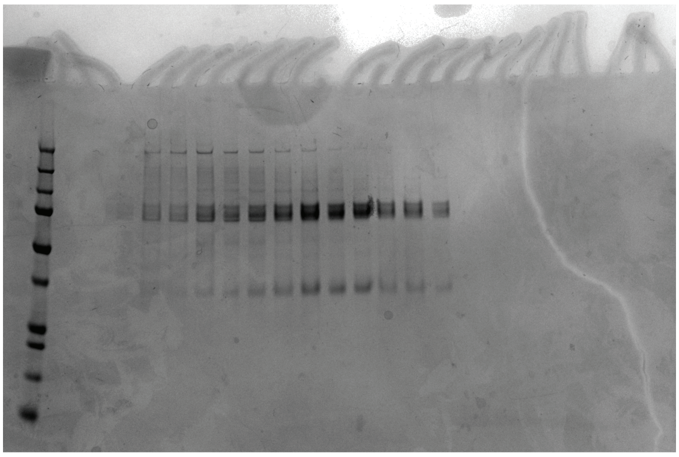

Source data for Supplementary Figure 1. Purification of MmCAT1, FrMLV-RBD, and MmCAT1:FrMLV-RBD complex. (**a**) Source data for Supplementary Figure 1b. (**b**) Source data for Supplementary Figure 1d. (**c**) Source data for Supplementary Figure 1h.
