# Supplementary material for: Amino acid and viral binding by the high-affinity Cationic Amino acid Transporter 1 (CAT1) from Mus musculus
Source: Nat Commun. 2026 Feb 16;17:2829. doi: 10.1038/s41467-026-69421-0 (PMC13022406; doi:10.1038/s41467-026-69421-0)
Supplement: Supplementary file 1 — Supplementary Information [file 41467_2026_69421_MOESM1_ESM.pdf]

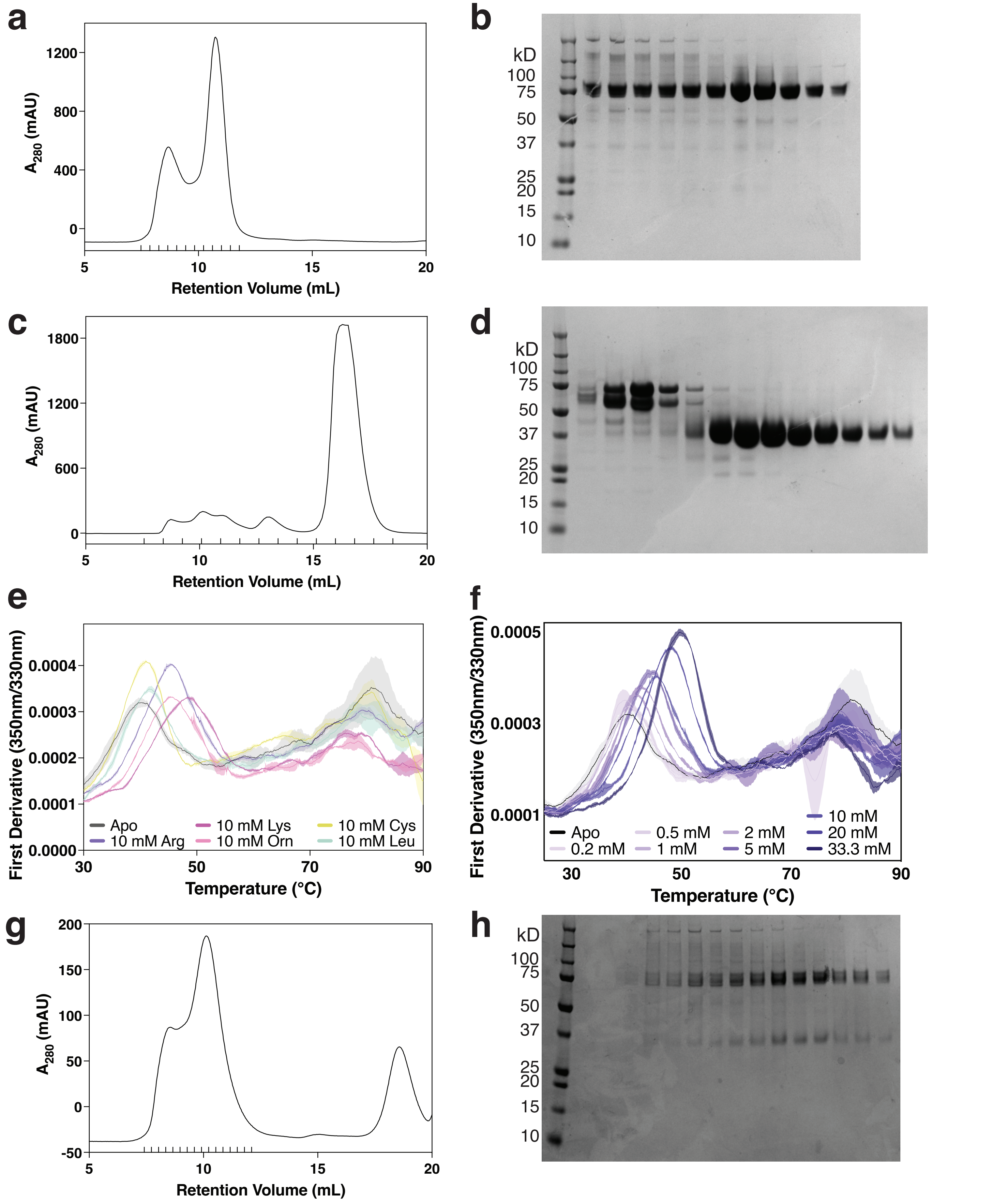


**Supplementary Figure 1. Purification of MmCAT1, FrMLV-RBD, and MmCAT1: FrMLV-RBD complex.** Purification of MmCAT1 by (**a**) preparative size-exclusion chromatography of MmCAT1 on a Superdex 200 Increase 10/300 GL column and (**b**) representative SDS-PAGE of MmCAT1 purification from 10 biological replicates. Purification of FrMLV-RBD by (**c**) preparative size-exclusion chromatography of MmCAT1 on a Superdex 200 Increase 10/300 GL column and (**d**) representative SDS-PAGE of MmCAT1 purification from 10 biological replicates. NanoDSF of MmCAT1 with (**e**) various amino acids and (**f**) various concentrations of arginine (N=3). Purification of MmCAT1:FrMLV-RBD by (**g**) preparative size-exclusion chromatography of MmCAT1 on a Superdex 200 Increase 10/300 GL column and (**h**) representative SDS-PAGE of MmCAT1 purification from 10 biological replicates.

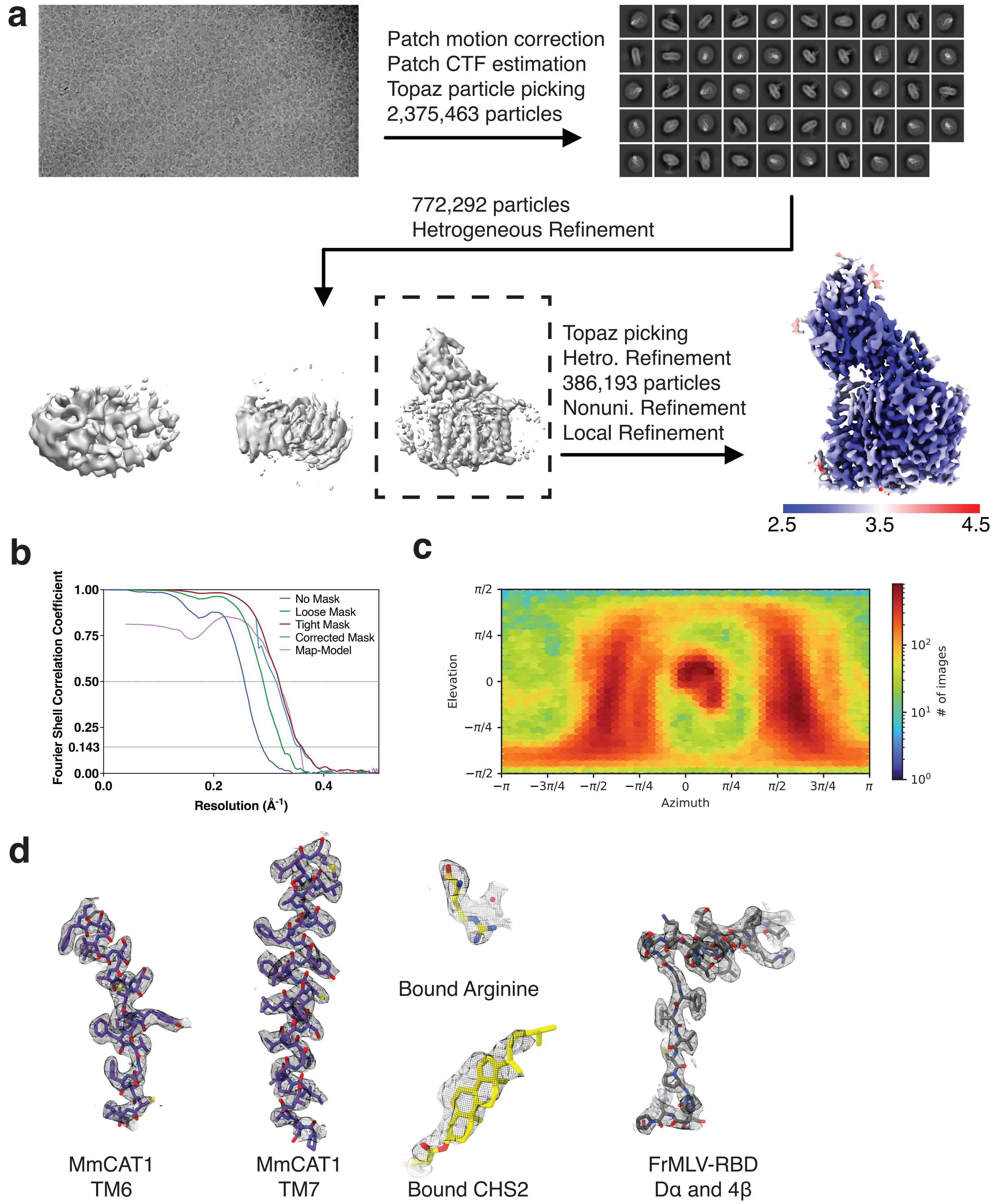


**Supplementary Figure 2. Structure determination of the Arginine bound MmCAT1: FrMLV-RBD complex.** (**a**) Flow chart for determining the structure of the Arginine bound MmCAT1:FrMLV-RBD complex. (**b**) Fourier shell correlation coefficient for cryo-EM map reconstruction and model refinement. (**c**) Particle orientation distribution for cryo-EM map reconstruction. (**d**) Representative density for MmCAT1, FrMLV-RBD, and bound arginine and cholesterol hemisuccinate (CHS).

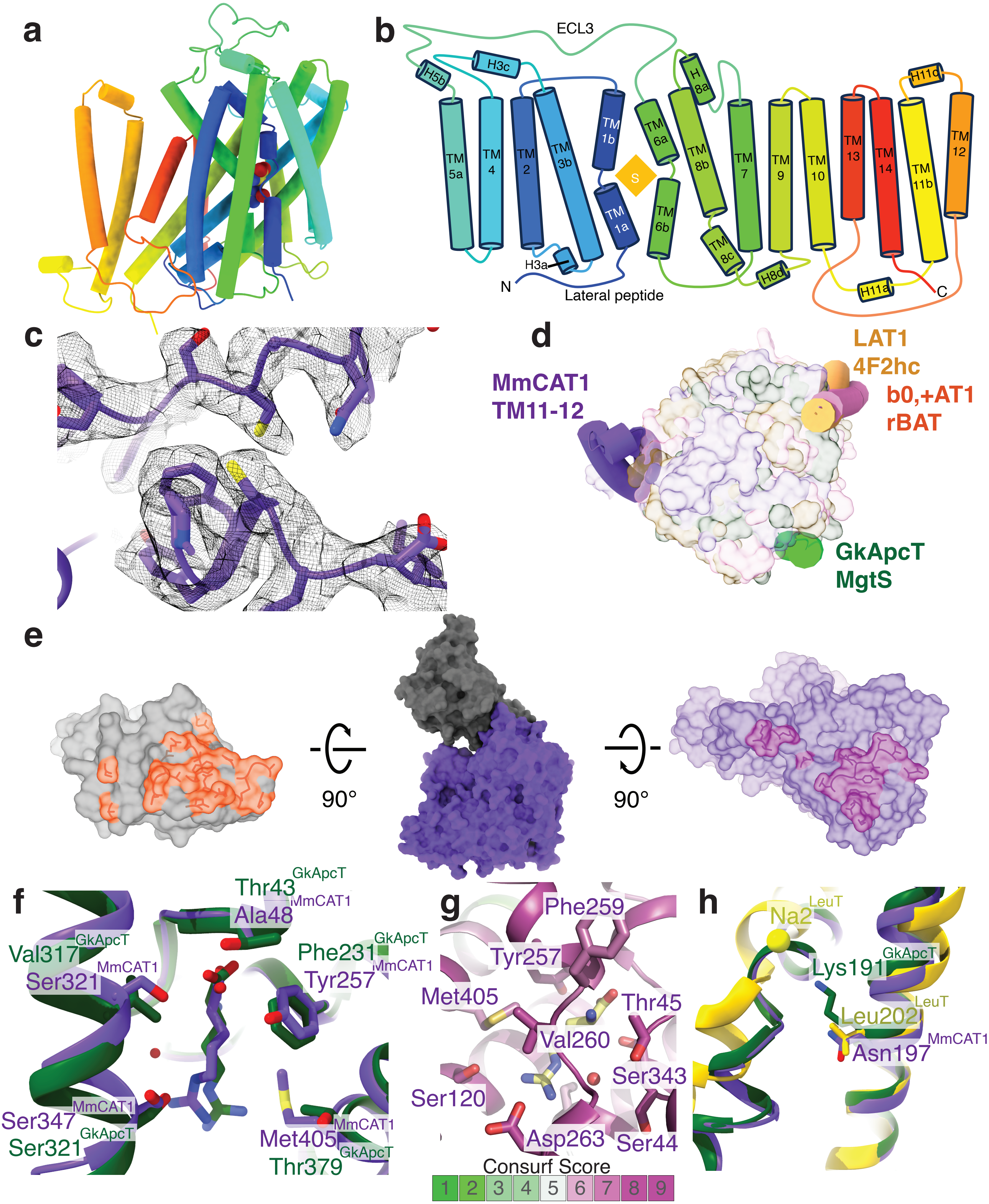


**Supplementary Figure 3. Structural analysis of MmCAT1:FrMLV-RBD.** (**a**) Secondary structure of MmCAT1. (**b**) Topology of MmCAT1. (**c**) Structure and density of MmCAT1's Cys226 and Cys309. (**d**) Structures of MmCAT1, GkApcT, B0,+AT1, and LAT1, aligned on the APC domain. (**e**) Open book diagram of MmCAT1:FrMLV-RBD complex interface. (**f**) Arginine binding of MmCAT1 and GkApcT, colored in purple and green, respectively. (**g**) Binding site conservation of MmCAT1 homologs. (**h**) Position of the Na2 site in LeuT, and the occupying Asn197 of MmCAT1 and Lys191 of GkApcT. MmCAT1, GkApcT, and LeuT are colored in purple, green, and yellow, respectively.

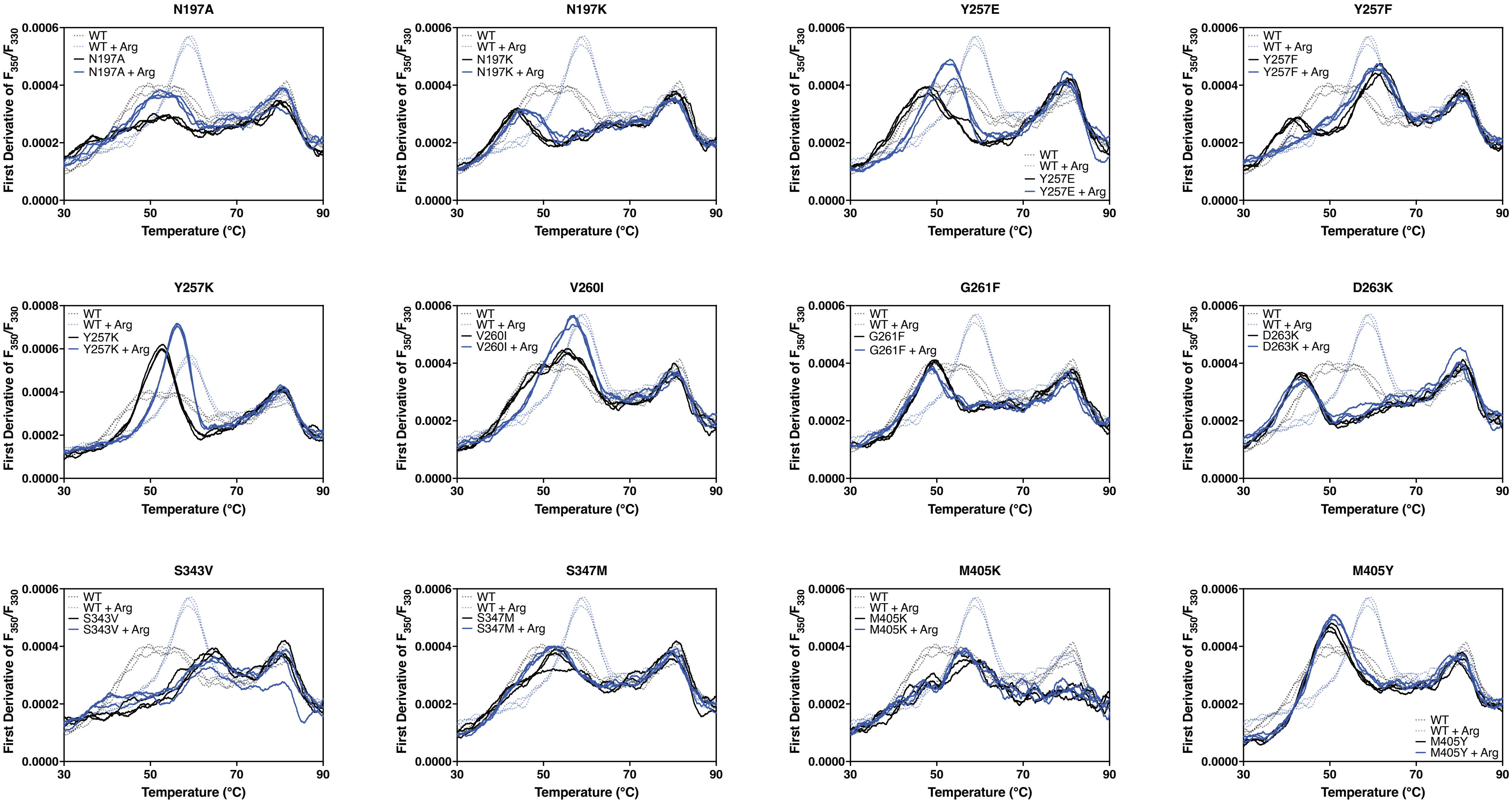


**Supplementary Figure 4. Thermostability of MmCAT1 mutants with and without arginine.** NanoDSF for each mutant, purified in LMNG/CHS, with and without ligand. Individual experiments are shown for each experiment of technical replicates (N=3) as solid lines and dotted lines for mutants and wild type protein, respectively.

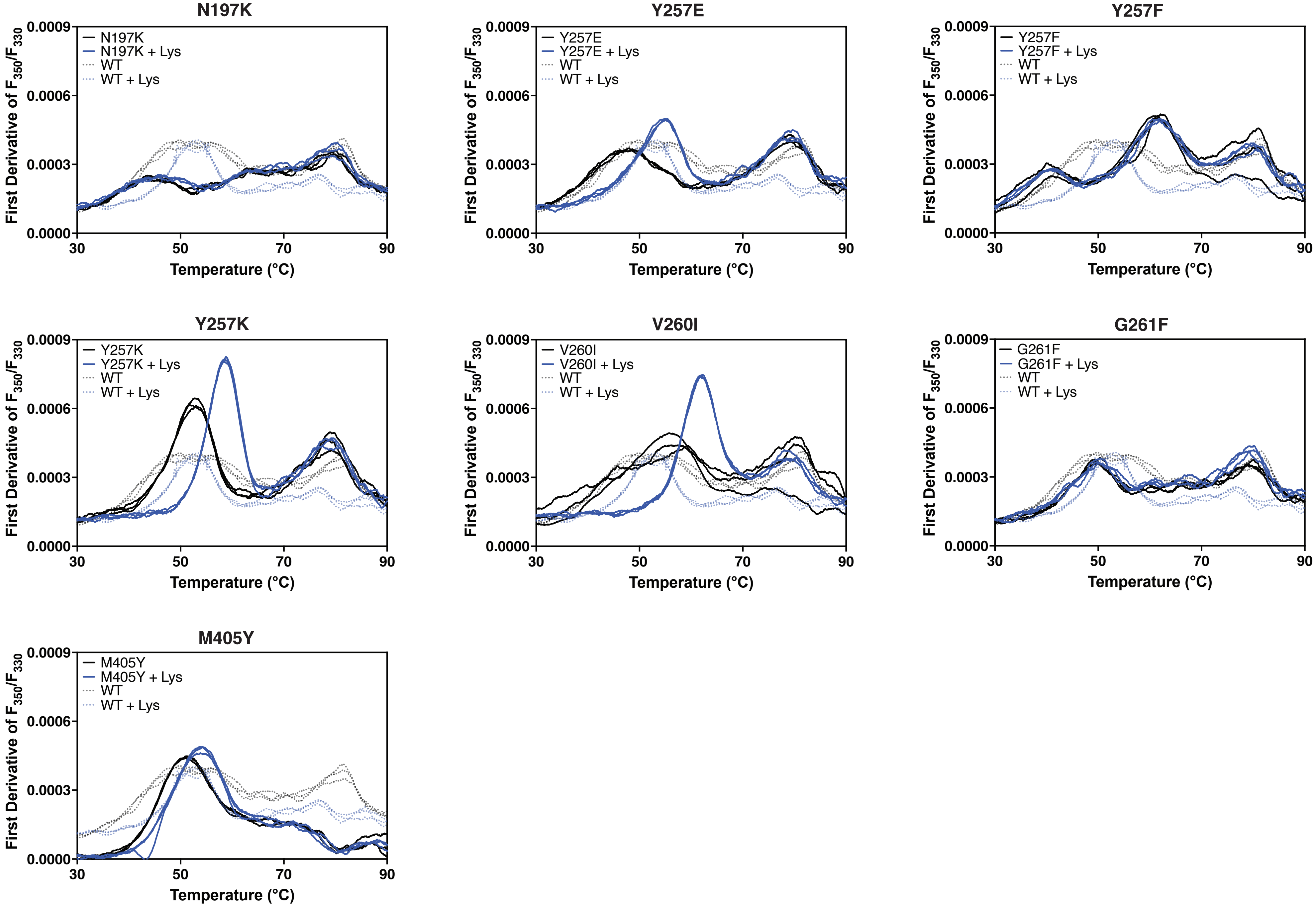


**Supplementary Figure 5. Thermostability of MmCAT1 mutants with and without lysine.** NanoDSF for each mutant, purified in LMNG/CHS, with and without ligand. Individual experiments are shown for each experiment of technical replicates (N=3) as solid lines and dotted lines for mutants and wild type protein, respectively.

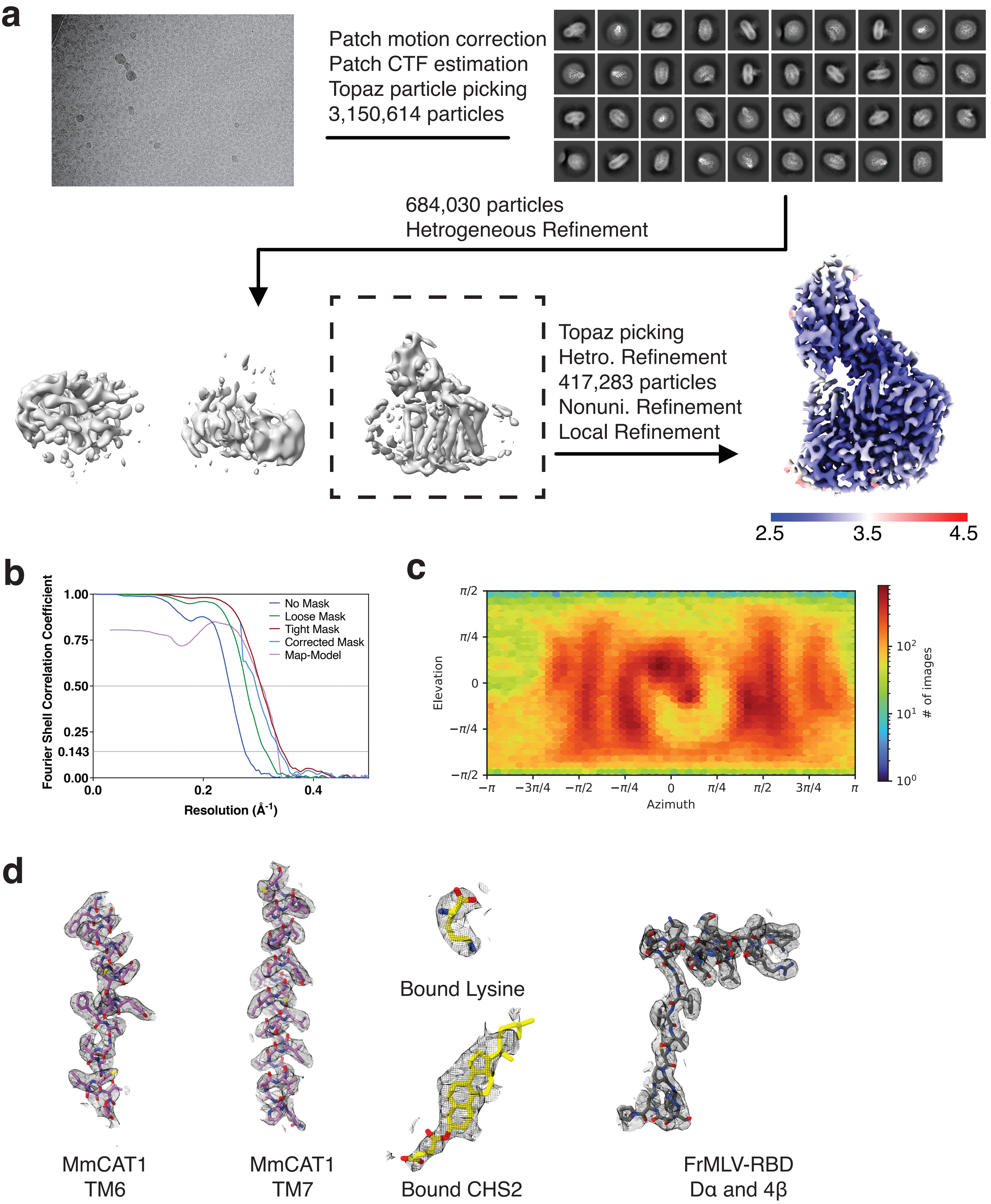


**Supplementary Figure 6. Structure determination of the lysine-bound MmCAT1:FrMLV-RBD complex.** (**a**) Flow chart for determining the structure of the lysine-bound MmCAT1:FrMLV-RBD complex. (**b**) Fourier shell correlation coefficient for cryo-EM map reconstruction and model refinement. (**c**) Particle orientation distribution for cryo-EM map reconstruction. (**d**) Representative density for MmCAT1, FrMLV-RBD, and bound lysine and cholesterol hemisuccinate (CHS).

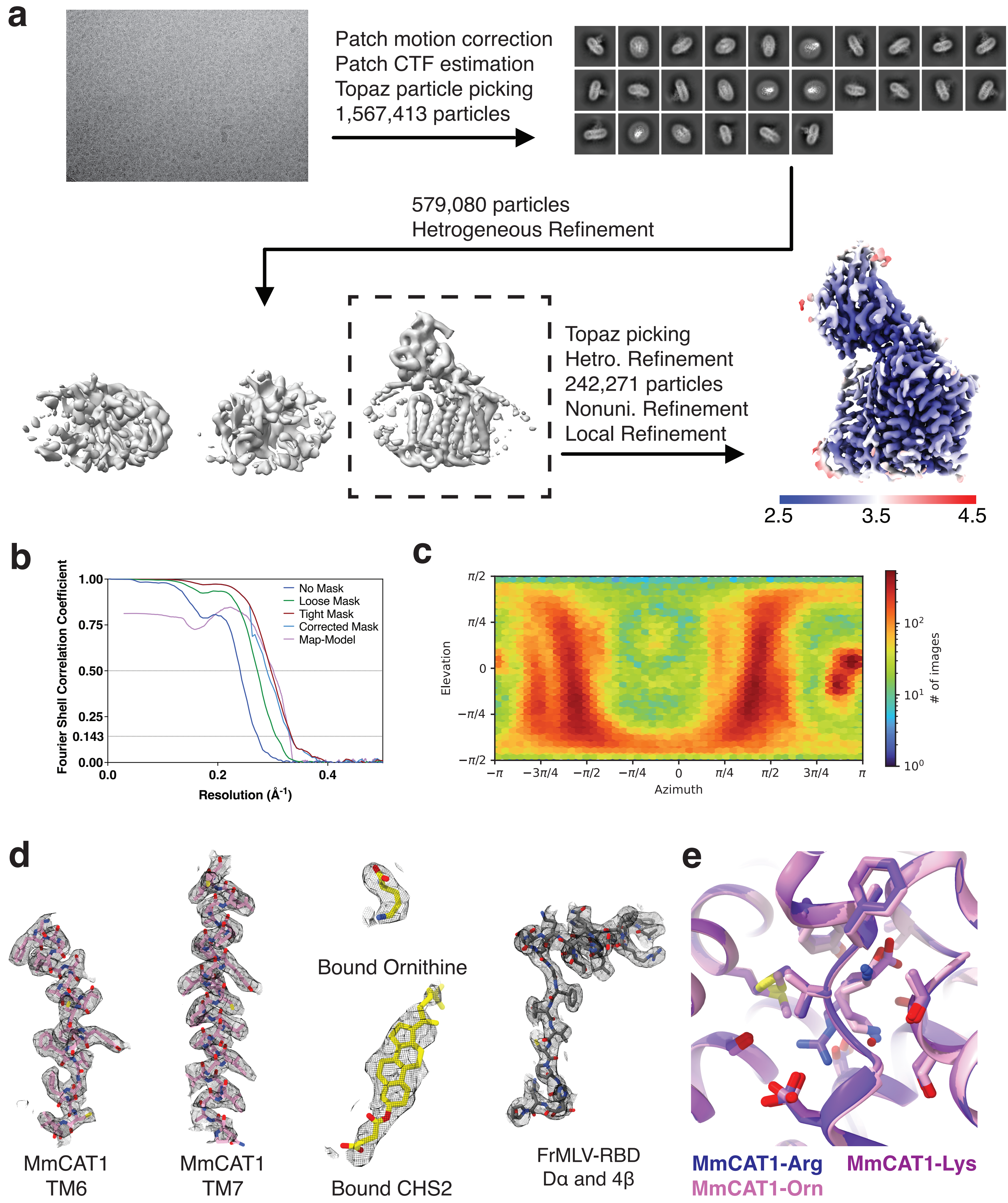


**Supplementary Figure 7. Structure determination of the ornithine-bound MmCAT1: FrMLV-RBD complex.** (**a**) Flow chart for determining the structure of the ornithine-bound MmCAT1:FrMLV-RBD complex. (**b**) Fourier shell correlation coefficient for cryo-EM map reconstruction and model refinement. (**c**) Particle orientation distribution for cryo-EM map reconstruction. (**d**) Representative density for MmCAT1, FrMLV-RBD, and bound ornithine and cholesterol hemisuccinate (CHS). (**e**) Overlay of MmCAT1 substrate binding site bound to arginine, lysine, and ornithine.

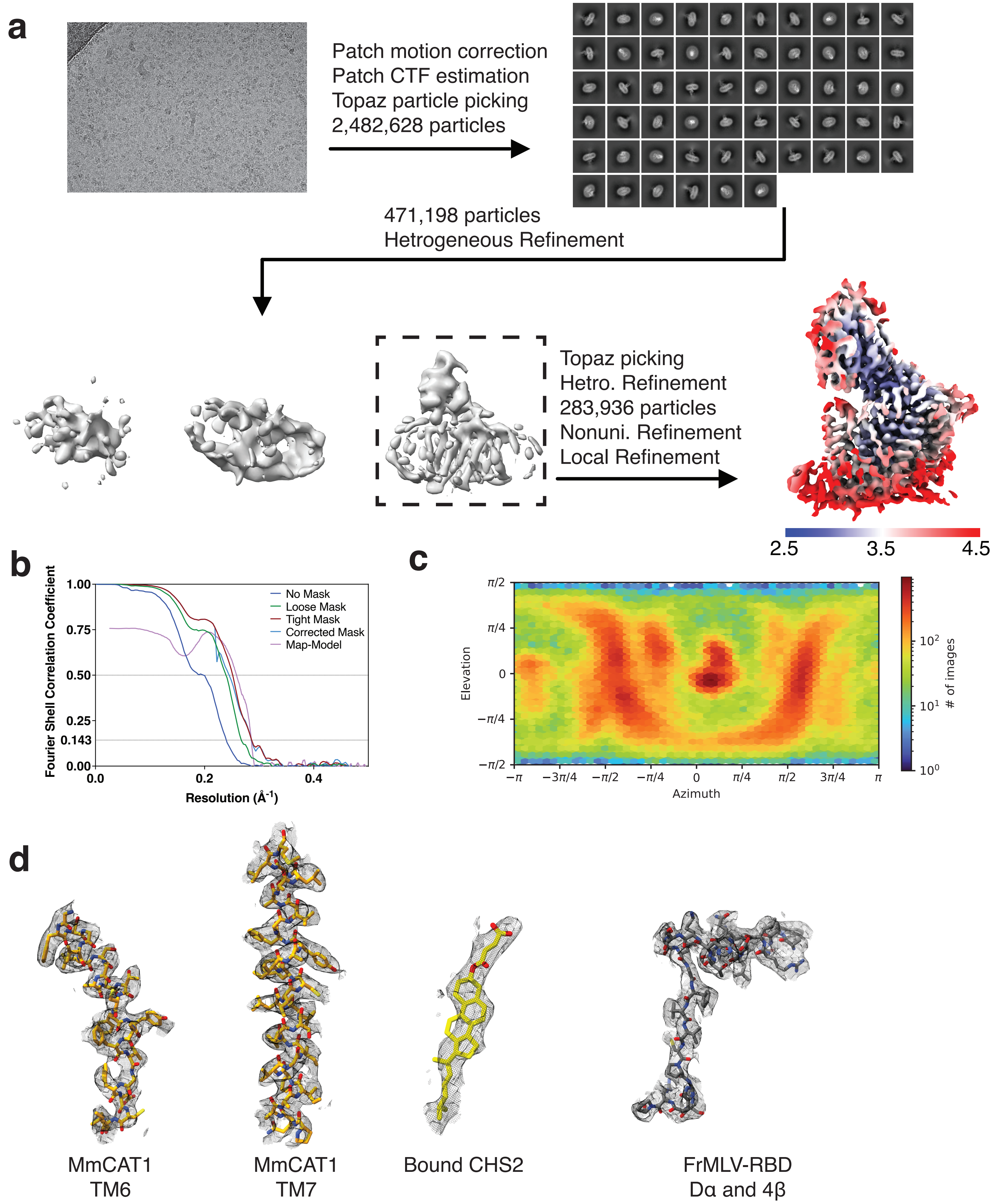


**Supplementary Figure 8. Structure determination of the apo MmCAT1:FrMLV-RBD complex.** (**a**) Flow chart for determining the structure of the substrate free MmCAT1:FrMLV-RBD complex. (**b**) Fourier shell correlation coefficient for cryo-EM map reconstruction and model refinement. (**c**) Particle orientation distribution for cryo-EM map reconstruction. (**d**) Representative density for MmCAT1, FrMLV-RBD, and bound cholesterol hemisuccinate (CHS).

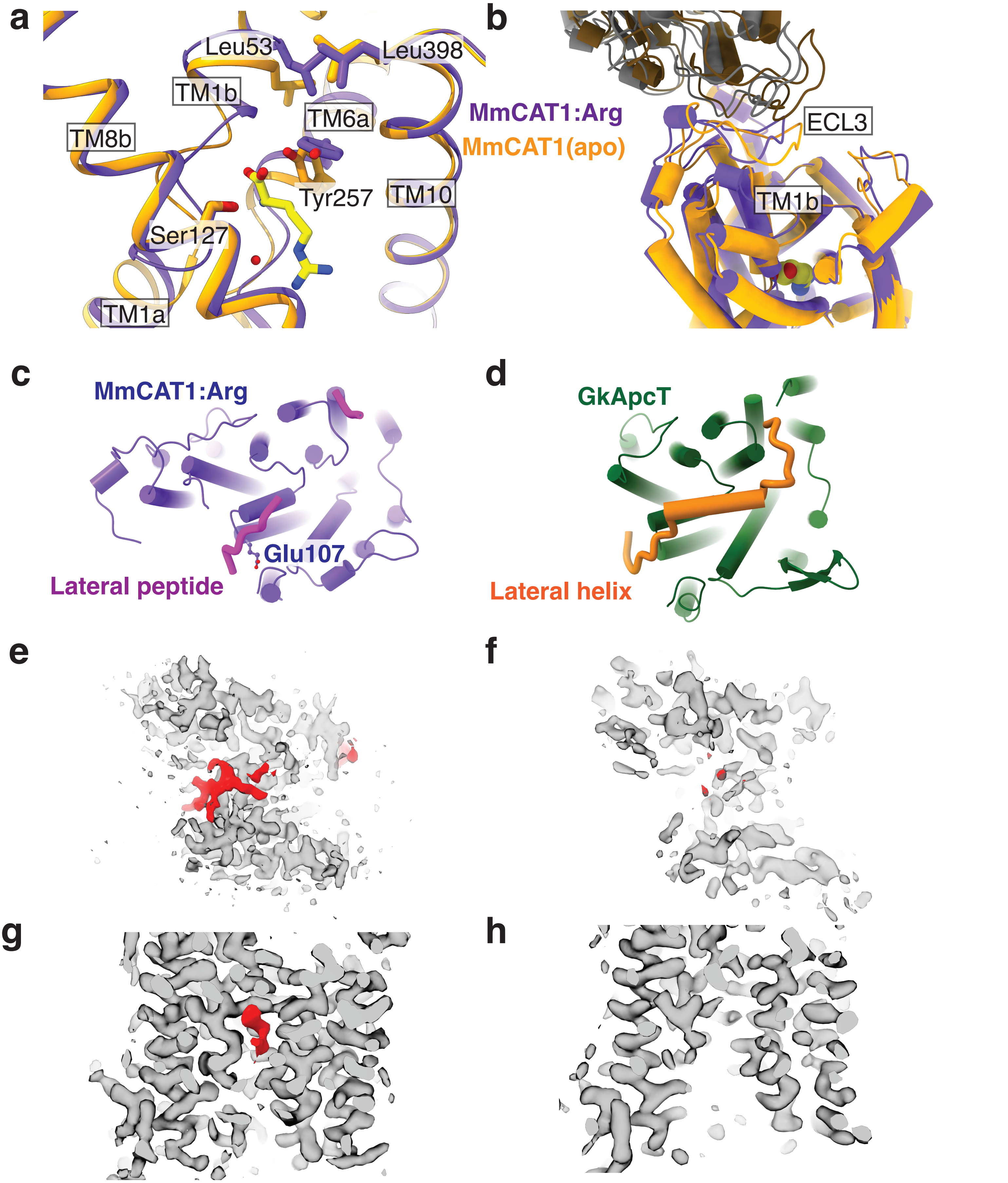


**Supplementary Figure 9. Structure comparison of substrate-bound and apo MmCAT1.** (**a**) Extracellular gate and (**b**) extracellular face of arginine-bound and apo MmCAT1, colored in purple and orange, respctively. Location of the lateral peptide (**c**) in MmCAT1 and (**d**) the equivalent position in GkApcT. Cryo-EM map of MmCAT1, with lateral peptide colored in red, for (**e**) arginine-bound and (**f**) apo state structures. Cryo-EM map of MmCAT1 with for (**g**) arginine-bound and (**h**) apo state structures, with arginine colored in red.

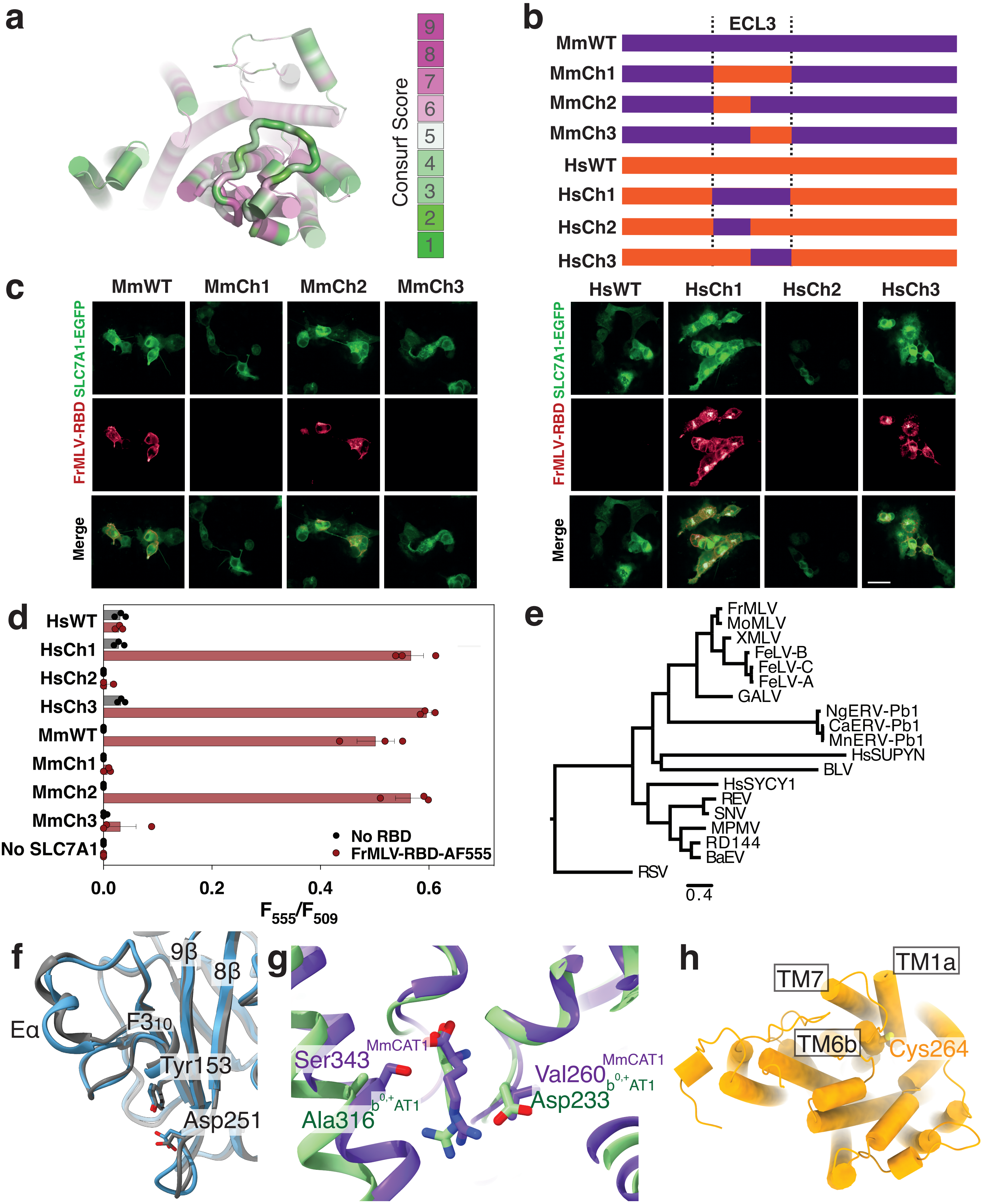


**Supplementary Figure 10. Viral binding of CAT1, orthoretrovirus RBDs, and their orthologs proteins.** (**a**) Sequence conservation of MmCAT1 homologs proteins. (**b**) Construct design of SLC7A1 ECL3 chimeras. (**c**) Confocal imaging and (**d**) normalized colocalization intensity of HEK293 cells expressing EGFP-tagged human and mouse SLC7A1 proteins, and ECL3 loop chimeras, stained with FrMLV-RBD-AF555, from 3 biological replicates. Scale bar is 50 µm. (**e**) Phylogenetic tree of orthoretrovirus envelope proteins. (**f**) Overlay of FrMLV-RBD bound to MmCAT1 and apo (PDB: 1AOL) colored in grey and blue, respectively. (**g**) Arginine binding of MmCAT1 and $b^{0,+}$AT1, colored in purple and green, respectively. (**h**) Location of Cys264 in apo MmCAT1.

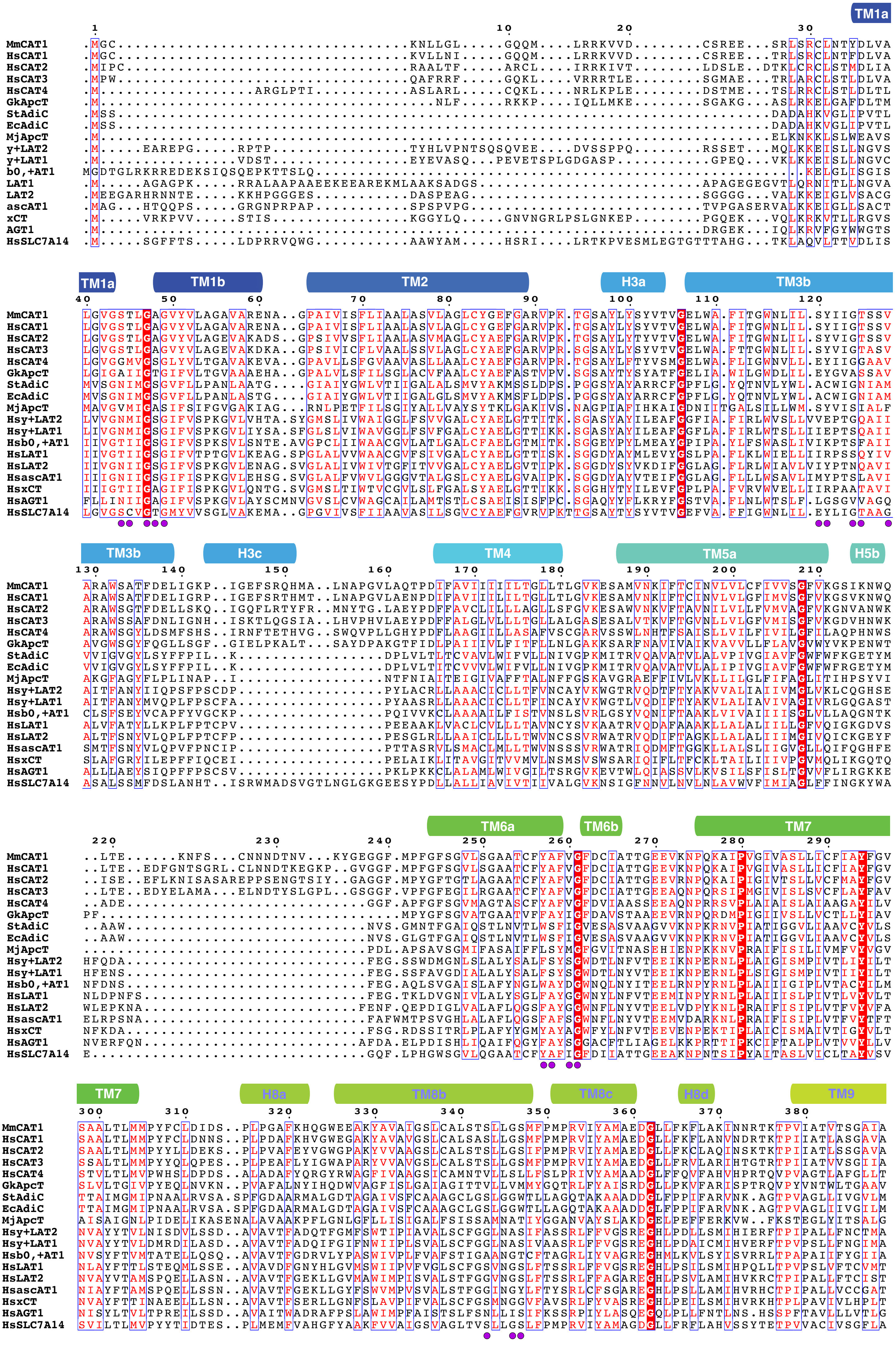

TM1a
1 10 20 30
MmCAT1 .MGC.............................KNLLGL.....GQQM....LRRKVVD........CSREE...SRLSRCLNTYDLVA
HsCAT1 .MGC.............................KVLLNI.....GQQM....LRRKVVD........CSREE...TRLSRCLNTFDLVA
HsCAT2 .MIPC............................RAALTF.....ARCL....IRRKIVT........LDSLE..DTKLCRCLSTMDLIA
HsCAT3 .MPW.............................QAFRRF.....GQKL....VRRRTLE........SGMAE...TRLARCLSTLDLVA
HsCAT4 .M..................ARGLPTI......ASLARL.....CQKL....NRLKPLE........DSTME...TSLRRCLSTLDLTL
GkApcT .M.......................................NLF.....RKKP....IQLLMKE........SGAKG...ASLRKELGAFDLTM
StAdiC .MSS.....................................................................DADAHKVGLIPVTL
EcAdiC .MSS.....................................................................DADAHKVGLIPVTL
MjApcT .M.......................................................................ELKNKKLSLWEAVS
y+LAT2 .M.....EAREPG.....RPTP...........TYHLVPNTSQSQVEE....DVSSPPQ........RSSET...MQLKKEISLLNGVS
y+LAT1 .M.....................VDST......EYEVASQ...PEVETSPLGDGASP............GPEQ...VKLKKEISLLNGVC
b0,+AT1 MGDTGLRKRREDEKSIQSQEPKTTSLQ.......................................................KELGLISGIS
LAT1 .M.....AGAGPK.....RRALAAPAAEEKEEAREKMLAAKSADGS...................APAGEGEGVTLQRNITLLNGVA
LAT2 .MEEGARHRNNTE.....KKHPGGGES......DASPEAG......................SGGGG...VALKKEIGLVSACG
ascAT1 .MAG....HTQQPS.....GRGNPRPAP......SPSPVPG.....................TVPGASERVALKKEIGLLSACT
xCT .M.....VRKPVV.....STIS...........KGGYLQ.....GNVNGRLPSLGNKEP........PGQEK...VQLKRKVTLLRGVS
AGT1 .M........................................................................DRGEK...IQLKRVFGYWWGTS
HsSLC7A14 .M.....SGFFTS.....LDPRRVQWG......AAWYAM.....HSRI....LRTKPVESMLEGTGTTTAHG...TKLAQVLTTVDLIS
TM1a TM1b TM2 H3a TM3b
40 50 60 70 80 90 100 110 120
MmCAT1 LGVGSTLGAGVYVLAGAVARENA..GPAIVISFLIAALASVLAGLCYGEFGARVPK.TGSAYLYSYVTVGELWA.FITGWNLIL.SYIIGTSSV
HsCAT1 LGVGSTLGAGVYVLAGAVARENA..GPAIVISFLIAALASVLAGLCYGEFGARVPK.TGSAYLYSYVTVGELWA.FITGWNLIL.SYIIGTSSV
HsCAT2 LGVGSTLGAGVYVLAGEVAKADS..GPSIVVSFLIAALASVMAGLCYAEFGARVPK.TGSAYLYTYVTVGELWA.FITGWNLIL.SYVIGTSSV
HsCAT3 LGVGSTLGAGVYVLAGEVAKDKA..GPSIVICFLVAALSSVLAGLCYAEFGARVPR.SGSAYLYSYVTVGELWA.FTTGWNLIL.SYVIGTASV
HsCAT4 LGVGGMVGSGLYVLTGAVAKEVA..GPAVLLSFGVAAVASLLAALCYAEFGARVPR.TGSAYLFTYVSMGELWA.FLIGWNVLL.EYIIGGAAV
GkApcT LGIGAIIGTGIFVLTGVAAAEHA..GPALVLSFILSGLACVFAALCYAEFASTVPV.SGSAYTYSYATFGELIA.WILGWDLIL.EYGVASSAV
StAdiC MVSGNIMGSGVFLLPANLASTG....GIAIYGWLVTIIGALALSMVYAKMSSLDPS.PGGSYAYARRCFGPFLG.YQTNVLYWL.ACWIGNIAM
EcAdiC MVSGNIMGSGVFLLPANLASTG....GIAIYGWLVTIIGALGLSMVYAKMSFLDPS.PGGSYAYARRCFGPFLG.YQTNVLYWL.ACWIGNIAM
MjApcT MAVGVMIGASIFSIFGVGAKIAG...RNLPETFILSGIYALLVAYSYTKLGAKIVS.NAGPIAFIHKAIGDNIITGALSILLWM.SYVISIALF
Hsy+LAT2 LVVGNMIGSGIFVSPKGVLVHTA.SYGMSLIVWAIGGLFSVVGALCYAELGTTITK.SGASYAYILEAFGGFIA.FIRLWVSLLVVEPTGQAII
Hsy+LAT1 LIVGNMIGSGIFVSPKGVLIYSA.SFGLSLVIWAVGGLFSVFGALCYAELGTTIKK.SGASYAYILEAFGGFLA.FIRLWTSLLIIEPTSQAII
Hsb0,+AT1 IIVGTIIGSGIFVSPKSVLSNTE.AVGPCLIIWAACGVLATLGALCFAELGTMITK.SGGEYPYLMEAYGPIPA.YLFSWASLIVIKPTSFAII
HsLAT1 IIVGTIIGSGIFVTPTGVLKEAG.SPGLALVVWAACGVFSIVGALCYAELGTTISK.SGGDYAYMLEVYGSLPA.FLKLWIELLIIRPSSQYIV
HsLAT2 IIVGNIIGSGIFVSPKGVLENAG.SVGLALIVWIVTGFITVVGALCYAELGVTIPK.SGGDYSYVKDIFGGLAG.FLRLWIAVLVIYPTNQAVI
HsascAT1 IIIGNIIGSGIFISPKGVLEHSG.SVGLALFVWVLGGGVTALGSLCYAELGVAIPK.SGGDYAYVTEIFGGLAG.FLLLWSAVLIMYPTSLAVI
HsxCT IIIGTIIGAGIFISPKGVLQNTG.SVGMSLTIWTVCGVLSLFGALSYAELGTTIKK.SGGHYTYILEVFGPLPA.FVRVWVELLIIRPAATAVI
HsAGT1 FLLINIIGAGIFVSPKGVLAYSCMNVGVSLCVWAGCAILAMTSTLCSAEISISFPC.SGAQYYFLKRYFGSTVA.FLNLWTSLF.LGSGVVAGQ
HsSLC7A14 LGVGSCVGTGMYVVSGLVAKEMA..GPGVIVSFIIAAVASILSGVCYAEFGVRVPKTTGSAYTYSYVTVGEFVA.FFIGWNLIL.EYLIGTAAG
TM3b H3c TM4 TM5a H5b
130 140 150 160 170 180 190 200 210
MmCAT1 ARAWSATFDELIGKP..IGEFSRQHMA..LNAPGVLAQTPDIFAVIIIIILTGLLTLGVKESAMVNKIFTCINVLVLCFIVVSGFVKGSIKNWQ
HsCAT1 ARAWSATFDELIGRP..IGEFSRTHMT..LNAPGVLAENPDIFAVIIILILTGLLTLGVKESAMVNKIFTCINVLVLGFIMVSGFVKGSVKNWQ
HsCAT2 ARAWSGTFDELLSKQ..IGQFLRTYFR..MNYTG.LAEYPDFFAVCLILLLAGLLSFGVKESAWVNKVFTAVNILVLLFVMVAGFVKGNVANWK
HsCAT3 ARAWSSAFDNLIGNH..ISKTLQGSIA..LHVPHVLAEYPDFFALGLVLLLTGLLALGASESALVTKVFTGVNLLVLGFVMISGFVKGDVHNWK
HsCAT4 ARAWSGYLDSMFSHS..IRNFTETHVG..SWQVPLLGHYPDFLAAGIILLASAFVSCGARVSSWLNHTFSAISLLVILFIVILGFILAQPHNWS
GkApcT AVGWSGYFQGLLSGF..GIELPKALT...SAYDPAKGTFIDLPAIIIVLFITFLLNLGAKKSARFNAVIVAIKVAVVLLFLAVGVWYVKPENWT
StAdiC VVIGVGYLSYFFPIL..K.................DPLVLTLTCVAVLWIFVLLNIVGPKMITRVQAVATVLALVPIVGIAVFGWFWFKGETYM
EcAdiC VVIGVGYLSYFFPIL..K.................DPLVLTITCVVVLWIFVLLNIVGPKMITRVQAVATVLALIPIVGIAVFGWFWFRGETYM
MjApcT AKGFAGYFLPLINAP..I.................NTFNIAITEIGIVAFFTALNFFGSKAVGRAEFFIVLVKLLILGLFIFAGLITIHPSYVI
Hsy+LAT2 AITFANYIIQPSFPSCDP..................PYLACRLLAAACICLLTFVNCAYVKWGTRVQDTFTYAKVVALIAIIVMGLVKLCQGHSE
Hsy+LAT1 AITFANYMVQPLFPSCFA..................PYAASRLLAAACICLLTFINCAYVKWGTLVQDIFTYAKVLALIAVIVAGIVRLGQGAST
Hsb0,+AT1 CLSFSEYVCAPFYVGCKP..................PQIVVKCLAAAAILFISTVNSLSVRLGSYVQNIFTAAKLVIVAIIIISGLVLLAQGNTK
HsLAT1 ALVFATYLLKPLFPTCPV..................PEEAAKLVACLCVLLLTAVNCYSVKAATRVQDAFAAAKLLALALIILLGFVQIGKGDVS
HsLAT2 ALTFSNYVLQPLFPTCFP..................PESGLRLLAAICLLLLTWVNCSSVRWATRVQDIFTAGKLLALALIIIMGIVQICKGEYF
HsascAT1 SMTFSNYVLQPVFPNCIP..................PTTASRVLSMACLMLLTWVNSSSVRWATRIQDMFTGGKLLALSLIIGVGLLQIFQGHFE
HsxCT SLAFGRYILEPFFIQCEI..................PELAIKLITAVGITVVMVLNSMSVSWSARIQIFLTFCKLTAILIIIVPGVMQLIKGQTQ
HsAGT1 ALLLAEYSIQPFFPSCSV..................PKLPKKCLALAMLWIVGILTSRGVKEVTWLQIASSVLKVSILSFISLTGVVFLIRGKKE
HsSLC7A14 ASALSSMFDSLANHT..ISRWMADSVGTLNGLGKGEESYPDLLALLIAVIVTIIVALGVKNSIGFNNVLNVLNLAVWVFIMIAGLFFINGKYWA
TM6a TM6b TM7
220 230 240 250 260 270 280 290
MmCAT1 ..LTE......KNFS...CNNNDTNV...KYGEGGF.MPFGFSGVLSGAATCFYAFVGFDCIATTGEEVKNPQKAIPVGIVASLLICFIAYFGV
HsCAT1 ..LTE..EDFGNTSGRL.CLNNDTKEGKP.GVGGF.MPFGFSGVLSGAATCFYAFVGFDCIATTGEEVKNPQKAIPVGIVASLLICFIAYFGV
HsCAT2 ..ISE..EFLKNISASAREPPSENGTSIY.GAGGF.MPYGFTGTLAGAATCFYAFVGFDCIATTGEEVRNPQKAIPIGIVTSLLVCFMAYFGV
HsCAT3 ..LTE..EDYELAMA...ELNDTYSLGPL.GSGGF.VPFGFEGILRGAATCFYAFVGFDCIATTGEEAQNPQRSIPMGIVISLSVCFLAYFAV
HsCAT4 ..ADE.........................GGF.APFGFSGVMAGTASCFYAFVGFDVIAASSEEAQNPRRSVPLAIAISLAIAAGAYILV
GkApcT PF.............................MPYGFSGVSTAGAATVFFAYIGFDAVSTAAEEVRNPQRDMPIGIVSLLVCTLLYIAV
StAdiC ..AAW.........................NVS.GMNTFGAIQSTLNVTLWSFIGVESASVAAGVVKNPKRNVPIATIGGVLIAAVCYVLS
EcAdiC ..AAW.........................NVS.GLGTFGAIQSTLNVTLWSFIGVESASVAAGVVKNPKRNVPIATIGGVLIAAVCYVLS
MjApcT ..........................PDL.APSAVSGMIFASAIFFLSYMGFGVITNASEHIENPKKNVPRAIFISILIVMFVYVGV
Hsy+LAT2 HFQDA.........................FEG.SSWDMGNLSLALYSALFSYSGWDTLNFVTEEIKNPERNLPLAIGISMPIVTLIYILT
Hsy+LAT1 HFENS.........................FEG.SSFAVGDIALALYSALFSYSGWDTLNYVTEEIKNPERNLPLSIGISMPIVTIIYILT
Hsb0,+AT1 NFDNS.........................FEG.AQLSVGAISLAFYNGLWAYDGWNQLNYITEELRNPYRNLPLAIIIGIPLVTACYILM
HsLAT1 NLDPNFS.......................FEG.TKLDVGNIVLALYSGLFAYGGWNYLNFVTEEMINPYRNLPLAIIISLPIVTLVYVLT
HsLAT2 WLEPKNA.......................FENF.QEPDIGLVALAFLQGSFAYGGWNFLNYVTEELVDPYKNLPRAIFISIPLVTFVYVFA
HsascAT1 ELRPSNA.......................FAFWMTPSVGHLALAFLQGSFAFSGWNFLNYVTEEMVDARKNLPRAIFISIPLVTFVYTFT
HsxCT NFKDA.........................FSG.RDSSITRLPLAFYYGMYAYAGWFYLNFVTEEVENPEKTIPLAICISMAIVTIGYVLT
HsAGT1 NVERFQN.......................AFDA.ELPDISHLIQAIFQGYFAYSGGACFTLIAGELKKPRTTIPKCIFTALPLVTVVYLLV
HsSLC7A14 E.............................GQF.LPHGWSGVLQGAATCFYAFIGFDIIATTGEEAKNPNTSIPYAITASLVICLTAYVSV
TM7 H8a TM8b TM8c H8d TM9
300 310 320 330 340 350 360 370 380
MmCAT1 SAALTLMMPYFCLDIDS..PLPGAFKHQGWEEAKYAVAIGSLCALSTSLLGSMFPMPRVIYAMAEDGLLFKFLAKINNRTKTPVIATVTSGAIA
HsCAT1 SAALTLMMPYFCLDNNS..PLPDAFKHVGWEGAKYAVAVGSLCALSASLLGSMFPMPRVIYAMAEDGLLFKFLANVNDRTKTPIIATLASGAVA
HsCAT2 SAALTLMMPYFCLDKDS..PLPVAFEYVGWGPAKYVVAAGSLCALSTSLLGSIFPMPRVIYAMAEDGLLFKCLAQINSKTKTPIIATLSSGAVA
HsCAT3 SSALTLMMPYYQLQPES..PLPEAFLYIGWAPARYVVAVGSLCALSTSLLGSMFPMPRVIYAMAEDGLLFRVLARIHTGTRTPIIATVVSGIIA
HsCAT4 STVLTLMVPWHSLDPDS..ALADAFYQRGYRWAGFIVAAGSICAMNTVLLSLLFSLPRIVYAMAADGLFFQVFAHVHPRTQVPVAGTLAFGLLT
GkApcT SLVLTGIVPYEQLNVKN..PVAFALNYIHQDWVAGFISLGAIAGITTVLLVMMYGQTRLFYAISRDGLLPKVFARISPTRQVPYVNTWLTGAAV
StAdiC TTAIMGMIPNAALRVSA.SPFGDAARMALGDTAGAIVSFCAAAGCLGSLGGWTLLAGQTAKAAADDGLFPPIFARVNKAGTPVAGLLIVGVLM
EcAdiC TTAIMGMIPNAALRVSA.SPFGDAARMALGDTAGAIVSFCAAAGCLGSLGGWTLLAGQTAKAAADDGLFPPIFARVNKAGTPVAGLIIVGILM
MjApcT AISAIGNLPIDELIKASENALAVAAKPFLGNLGFLLISIGALFSISSAMNATIYGGANVAYSLAKDGELPEFFERKVW..FKSTEGLYITSALG
Hsy+LAT2 NVAYYTVLNISDVLSSD..AVAVTFADQTFGMFSWTIPIAVALSCFGGLNASIFASSRLFFVGSREGHLPDLLSMIHIERFTPIPALLFNCTMA
Hsy+LAT1 NVAYYTVLDMRDILASD..AVAVTFADQIFGIFNWIIPLSVALSCFGGLNASIVAASRLFFVGSREGHLPDAICMIHVERFTPVPSLLFNGIMA
Hsb0,+AT1 NVSYFTVMTATELLQSQ..AVAVTFGDRVLYPASWIVPLFVAFSTIGAANGTCFTAGRLIYVAGREGHMLKVLSYISVRRLTPAPAIIFYGIIA
HsLAT1 NLAYFTTLSTEQMLSSE..AVAVDFGNYHLGVMSWIIPVFVGLSCFGSVNGSLFTSSRLFFVGSREGHLPSILSMIHPQLLTPVPSLVFTCVMT
HsLAT2 NVAYVTAMSPQELLASN..AVAVTFGEKLLGVMAWIMPISVALSTFGGVNGSLFTSSRLFFAGAREGHLPSVLAMIHVKRCTPIPALLFTCIST
HsascAT1 NIAYFTAMSPQELLSSN..AVAVTFSERLLGYFSWVMPVSVALSTFGGSNGGVFTYSRLCFSGAREGHLPSLLAMIHVRHCTPLPALLVCCGAT
HsxCT NVAYYTTINAEELLLSN..AVAVTFSERLLGNFSLAVPIFVALSCFGSMNGGVFAVSRLFYVASREGHLPEILSMIHVRKHTPLPAVIVLHPLT
HsAGT1 NISYLTVLTPREILSSD..AVAITWADRAFPSLAWIMPFAISTSLFSNLLISIFKSSRPIYLASQEGQLPLLFNTLNS.HSSPFTAVLLVTLG
HsSLC7A14 SVILTLMVPYYTIDTES..PLMEMFVAHGFYAAKFVVAIGSVAGLTVSLLGSLFPMPRVIYAMAGDGLLFRFLAHVSSYTETPVVACIVSGFLA

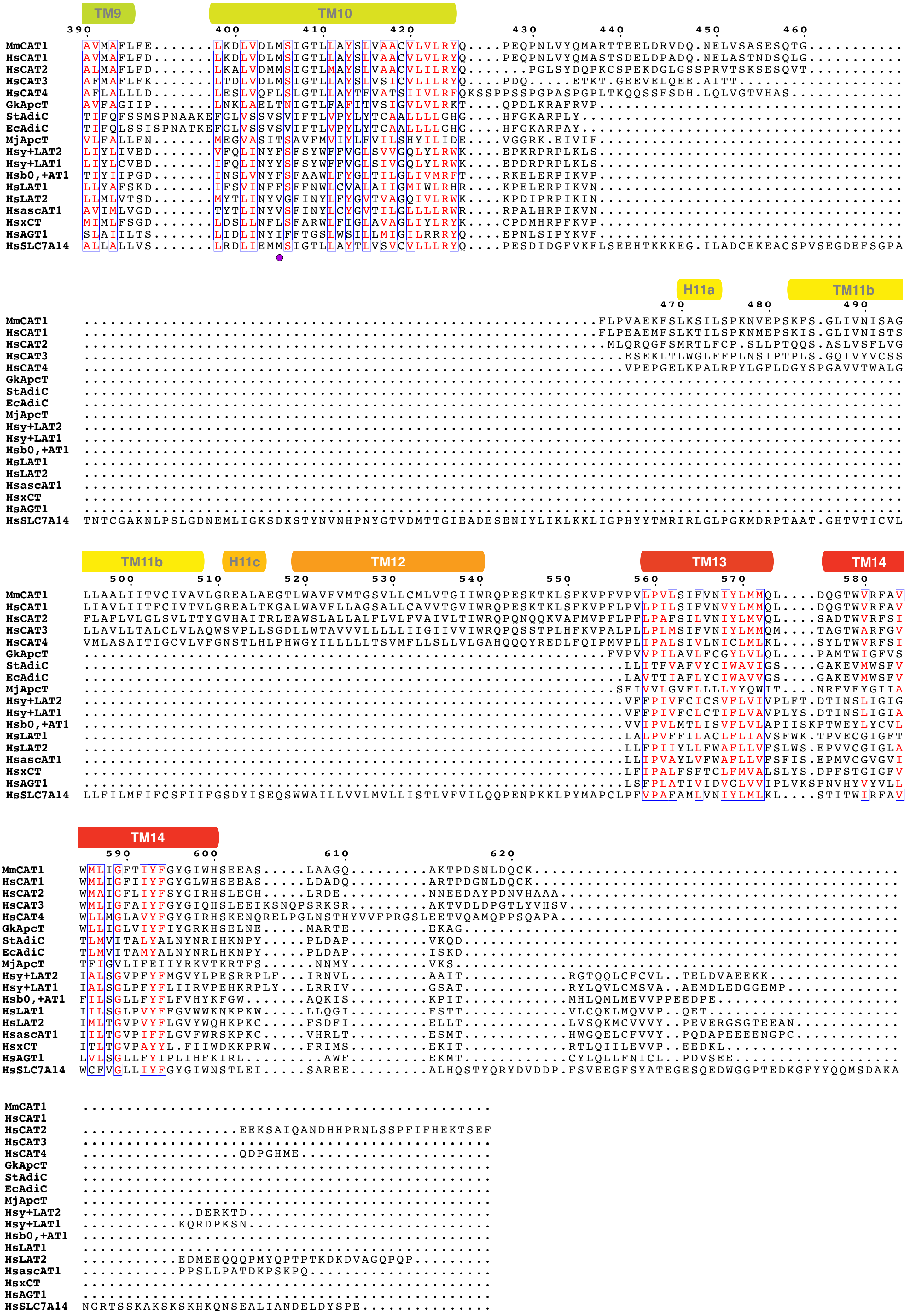


**Supplementary Figure 11.** Sequence alignment of MmCAT1 with bacterial and human homologs. Purple circles below the alignment indicate residues within 4Å of the arginine substrate.

**Supplementary Table 1. ECL3 sequences of chimeric SLC7A1 proteins**

| Chimera Construct | Background SLC7A1 gene | ECL3 sequence |
|---|---|---|
| MmWT | MmSLC7A1 | QLTE---KNFS---CNNNDT-NVKYGEGGFMPF |
| MmCh1 | MmSLC7A1 | QLTEEDFGNTSGRLCLNNDTKEGKPGVGGFMPF |
| MmCh2 | MmSLC7A1 | QLTEEDFGNTSGRLCNNNDT-NVKYGEGGFMPF |
| MmCh3 | MmSLC7A1 | QLTE---KNFS---CLNNDTKEGKPGVGGFMPF |
| HsWT | HsSLC7A1 | QLTEEDFGNTSGRLCLNNDTKEGKPGVGGFMPF |
| HsCh1 | HsSLC7A1 | QLTE----KNFS--CNNNDT-NVKYGEGGFMPF |
| HsCh2 | HsSLC7A1 | QLTE----KNFS--CLNNDTKEGKPGVGGFMPF |
| HsCh3 | HsSLC7A1 | QLTEEDFGNTSGRLCNNNDT-NVKYGEGGFMPF |

**Supplementary Table 2. Analysis sequence for co-localization of MmCAT1 and FrMLV-RBD in Harmony**

| Steps | Building block | Method | Input | Output |
|---|---|---|---|---|
| 1 | Input image | Flatfield correction: advanced<br>Brightfield correction: yes<br>Stack Processing: Individual plane<br>Create Global Image: No | Channel: Alexa 568<br>Channel: Alexa 488 | Output image |
| 2 | Find image region | Method: Absolute Threshold<br>Lowest intensity: ≥ 1500<br>Highest intensity: INF<br>Split into Objects: No | Channel: Alexa 488<br>ROI population: None | Output population: MmCAT1 |
| 3 | Find image region | Method: Absolute Threshold<br>Lowest intensity: ≥ 3500<br>Highest intensity: INF<br>Split into Objects: No | Channel: Alexa 568<br>ROI population: None | Output population: FrMLV-RBD |
| 4 | Calculate Intensity Properties | Method: Standard-Mean | Channel: Alexa 488<br>ROI population: MmCAT1 | Output property: MmCAT1 intensity |
| 5 | Calculate Intensity Properties | Method: Standard-Mean | Channel: Alexa 568<br>ROI population: FrMLV-RBD | Output property: FrMLV-RBD intensity |
| 6 | Calculate Morphology Properties | Method: Standard-Area ($\mu m^2$) | Channel: Alexa 568<br>ROI population: FrMLV-RBD | Output property: FrMLV-RBD area |
| 7 | Define results | Formula output: a/b<br>a = MmCAT1 intensity<br>b = FrMLV-RBD intensity | N/A | Output:<br>1. a/b<br>2. MmCAT1 intensity<br>3. FrMLV-RBD intensity<br>4. FrMLV-RBD area |
